# Spatio-temporal requirements for transposable element piRNA-mediated silencing during *Drosophila* oogenesis

**DOI:** 10.1093/nar/gkt1184

**Published:** 2013-11-27

**Authors:** Jérémy Dufourt, Cynthia Dennis, Antoine Boivin, Nathalie Gueguen, Emmanuelle Théron, Coline Goriaux, Pierre Pouchin, Stéphane Ronsseray, Emilie Brasset, Chantal Vaury

**Affiliations:** ^1^Inserm, UMR1103, F-63001 Clermont-Ferrand, France, ^2^CNRS, UMR6293, F-63001 Clermont-Ferrand, France, ^3^Clermont Université, Université d'Auvergne, Laboratoire GReD, BP 10448, F-63000 Clermont-Ferrand, France, ^4^Laboratoire Biologie du Développement, UMR7622, CNRS-Université Pierre et Marie Curie, 9 quai Saint Bernard, 75005 Paris, France and ^5^CHU, F-63001 Clermont-Ferrand, France

## Abstract

During *Drosophila* oogenesis, transposable element (TE) repression involves the Piwi-interacting RNA (piRNA) pathway which ensures genome integrity for the next generation. We developed a transgenic model to study repression of the *Idefix* retrotransposon in the germline. Using a candidate gene KD-approach, we identified differences in the spatio-temporal requirements of the piRNA pathway components for piRNA-mediated silencing. Some of them (Aub, Vasa, Spn-E) are necessary in very early stages of oogenesis within the germarium and appear to be less important for efficient TE silencing thereafter. Others (Piwi, Ago3, Mael) are required at all stages of oogenesis. Moreover, during early oogenesis, in the dividing cysts within the germarium, *Idefix* anti-sense transgenes escape host control, and this is associated with very low *piwi* expression. Silencing of *P*-element-based transgenes is also strongly weakened in these cysts. This region, termed the ‘Piwiless pocket’ or Pilp, may ensure that new TE insertions occur and are transmitted to the next generation, thereby contributing to genome dynamics. In contrast, piRNA-mediated silencing is strong in germline stem cells in which TE mobilization is tightly repressed ensuring the continued production of viable germline cysts.

## INTRODUCTION

Transposable element (TE) activity represents a constant threat for the stability of eukaryotic genomes and as a result protection mechanisms have evolved that limit TE mobilization. Nevertheless, TEs have colonized genomes efficiently and are thus thought to provide evolutionary advantages through their effects on genome expression and dynamics. Therefore, TEs should be able to bypass host defense mechanisms and mobilize in cells that will ensure their propagation to the next generation. Oogenesis is thus an important stage during which an active host defense is required to protect germline integrity, while escape from this protection can be positive from an evolutionary point of view. Extensive studies performed in *Drosophila* and mice have identified the piRNA pathway (PIWI-interacting RNA pathway) that requires small RNAs associated with PIWI proteins, the piRNAs, as the major pathway for silencing TEs in the germline and thereby inhibiting their mobilization and transmission (1–3). TE silencing by the piRNA pathway occurs both transcriptionally (TGS for Transcriptional Gene Silencing) and post-transcriptionally (PTGS for Post-Transcriptional Gene Silencing) (2,4–7). TGS involves decreased RNA synthesis due to the formation of a compact chromatin structure at target promoters, and PTGS involves homology-dependent target RNA degradation by PIWI-RISCs (PIWI-RNA-Induced Silencing Complexes).

Much of our knowledge about the piRNA pathway comes from studies of *Drosophila* oogenesis. In *Drosophila*, an ovary consists of 15 to 18 ovarioles, each of which contain a series of egg chambers at progressively advanced stages of oogenesis (8). The germline and somatic stem cells (GSC and SSC, respectively) reside in a region called the germarium at the anterior tip of each ovariole (9). During GSC division, one daughter cell remains in a ‘niche’ and continues to divide as a GSC. The other daughter cell, called the cystoblast, undergoes four cycles of mitotic division to form interconnected cysts of successively 2, 4, 8 and 16 germ cells. When the mature egg chamber leaves the germarium, the germline cyst consists of the oocyte and 15 nurse cells, all surrounded by a monolayer of somatic follicle cells deriving from the SSC. Both germ cells and follicle cells in the *Drosophila* ovary possess a functional piRNA pathway, which differs, however, in piRNA biogenesis. In both types of cells, a pool of primary piRNAs is processed from putative long single-stranded transcripts containing sequences homologous to TEs. These long transcripts are produced from discrete genomic loci (piRNA clusters), which reside primarily in pericentric heterochromatin enriched in TEs or their relics. Only in the germline, do these primary piRNAs, which include transposon anti-sense transcripts, target transposon sense-transcripts resulting in the production of a secondary pool of piRNAs (2). Secondary sense piRNAs enhance cleavage of anti-sense piRNA precursors, which leads to amplification of piRNA production called the ping-pong cycle. The three Argonaute proteins—Piwi, Aub and Ago3—are major players in this pathway and have also been shown to play a crucial role in gonadal development. Other factors, in particular components of the germ cell perinuclear structure, called the nuage, such as Vasa (Vas), Maelstrom (Mael), Armitage (Armi) and Squash (Squ), have also been implicated in piRNA production and transposon repression (10–14).

Our current knowledge of the piRNA pathway has been mostly deduced from high-throughput sequencing and large-scale genetic screens. They clearly demonstrated the existence of different classes of TEs undergoing differential regulation (2,4,5,15). Thus, the challenge now is to perform functional analyses on specific TEs to uncover the underlying specificities in their silencing or alternatively to determine what allows them to escape from silencing. The silencing of the retrotransposon *Idefix* has been previously characterized in ovarian follicle cells where its promoter is active; it has been reported to be repressed by the *flamenco* piRNA cluster (also called the COM locus) in a Piwi-dependent manner (16). In other somatic tissues, *Idefix* was shown to be repressed in a Piwi-independent manner by a transcriptional silencing pathway involving Polycomb group proteins (Pc-G) (17). In the germline, *Idefix* transcripts were found to be significantly upregulated in *piwi* germline knockdown ovaries (5,15). Several studies have also reported that piRNAs with a ping-pong signature and homologous to *Idefix* are produced in the germline and that they are reduced in *piwi* knockdown ovaries (15). These results suggest that a repression capacity exists for this TE in the germline. Here, we report that *Idefix*-sensor transgenes, whose expression can be induced in the germline, are targets of the piRNA pathway. Our data show that two categories of piRNA pathway proteins with different temporal requirements are involved in the silencing of *Idefix* sequences. In addition, we identified a small developmental window, corresponding to dividing germline cysts in the germarium, during which piRNA-mediated silencing is strongly reduced. Spatio-temporal regulation of TEs may contribute to the balance between TE repression and mobilizaton in the germline.

## MATERIALS AND METHODS

### *Drosophila* strains and transgenic lines

All experiments were performed at 22°C except when indicated. The fly strains *Act5C-Gal4* (3954 and 4414) and *nos-Gal4* (4937) came from the Bloomington stock center as did the RNAi lines *ago3* (35232), *aub* (35201), *mael* (35202), *spn-E* (35303), *piwi* (33724), *w* (35573, 33644), *vasa* (34950). *αtub-Gal4* were kind gifts from V. Mirouse, *piwi^NT^* from J. Brennecke.

The *Idefix*-sensor transgenic constructions were generated by inserting 419 bp of the *Idefix* gag coding region (1003–1422) in either the sense (*pGgIds*) or anti-sense (*pGgIdas*) orientation with respect to *gfp* transcription within the *UASp-gfp* vector. Six independent transgenic lines were generated for *pGgIds* and *pGgIdas*. *BC69* (kindly provided by JL Couderc) is a *P-lacZ* enhancer trap line (18) and contains an in-frame translational fusion of the *Escherichia coli lacZ* gene to the second exon of the *P* transposase gene and a *rosy* transformation marker (FBtp0000154). *RS3* is a *P-FRT-white* transgene (FBms0003945). It is inserted in the Telomeric Associated Sequences (TAS) of the 3R chromosomal arm (site 100E3). It is homozygous viable and fertile (Bloomington #123282). Crosses involving *BC69* were performed at 25°C.

The *pTomato-piwi* plasmid is the result of the LR clonase II reaction (Invitrogen) between on one hand *pUASp Tomato N term Gateway* (Kind gift from V. Mirouse), constructed by the initial insertion of *tomato* coding sequence (1431 bp; Genbank number AY678269.1) between KpnI and XhoI restriction sites of *pUASp* vector followed by the insertion of Gateway Cassette (Invitrogen) between BglII and SpeI restriction sites, and on the other hand *pDONR piwi* Gateway plasmid, obtained by BP clonase II reaction (Invitrogen) between *pDONR221* (Invitrogen) and PCR fragment amplified from *piwi* cDNA-containing pBluscript_SK(−) vector (Genbank number BT011138) using primers attB1_Piwi_F: GGG GAC AAG TTT GTA CAA AAA AGC AGG CTT GGC TGA TGA TCA GGG ACG T and attB2_Piwi_R GGG GAC CAC TTT GTA CAA GAA AGC TGG GTT TAT AGA TAA TAA AAC TTC TTT TC. *UASp-tomato-piwi* was introduced into the *Drosophila* genome using standard *P*-element-mediated transformation techniques at the Fly Facility platform (www.flyfacility.fr).

### Fluorescent *in situ* hybridization

A *gfp* fragment was cloned into *pGemt*-easy (Promega) using the following primers *gfp*-probe_for: 5′-TAGATGGTGATGTTAATGGGC-3′ and *gfp*-probe_rev: 5′-GTTTGTATAGTTCATCCATGCC-3′. RNA-FISH was performed as described in (19). Briefly, ovaries were dissected in PBT (PBS-0.2% Tween) on ice, fixed with 4% formaldehyde/PBT at room temperature (RT) for 10 min and rinsed three times with PBT. After permeabilization (1 h in PBS-0.3% Triton) prehybridization was carried out as follows: 10 min in HYB-(Formamide 50%, SSC 5×, Tween 0.02%)/PBT 1:1, 10 min in HYB-, 1 h in HYB+ (HYB- with yeast tRNA 0.1 μg/μl, heparin 0.25 mg/ml) at 60°C. Hybridization was carried out overnight at 60°C with 1 μg of RNA UTP-Dig-labeled probe. Ovaries were rinsed in HYB- and HYB-/PBT at 60°C then four times in PBT at RT. Blocking was done for 1 h at RT with TNB 0.3% triton (Perkin-Elmer TSA kit) and immunodetection 1.5 h at RT with anti-Dig-HRP (Roche) in TNB 0.2% tween. Ovaries were rinsed three times in PBT, incubated for 10 min with TSA-Cy3 in 1/25 amplification diluent (Perkin-Elmer), then rinsed three times and stained with DAPI (1/10 000). For RNA visualization, RNaseH treatment for 30 min at 37°C was performed before TSA amplification to destroy RNA/DNA hybrids.

### Immunostaining

Ovaries were dissected in PBS on ice, fixed with 4% formaldehyde/PBS at RT for 15 min and rinsed three times with PBT (PBS with 0.2%Triton X-100). After blocking in BBT (PBT with 0.1% BSA) for 2 h at RT, ovaries were incubated with primary antibodies in BBT overnight at 4°C. After three washes in PBT, ovaries were incubated with secondary antibodies for 2 h at RT. The primary antibodies used were α-Aub and α-Ago3 [1:500, rabbit; a kind gift from J. Brennecke (2)], α-Piwi P3G11 [directed against Piwi N-term; 1:1000, mouse; a kind gift from MC Siomi (20)], α-Piwi (ab5207Abcam rabbit polyclonal antibody obtained against the peptide corresponding to amino acids 350 to 450 in the Piwi protein), α-HP1 (1:100, mouse C1a9 from Developmental Studies Hybridoma Bank), α-Vasa (1:100, rat from Developmental Studies Hybridoma Bank, DSHB), Rhino (1:1000, guinea pig from a kind gift from P. Zamore), αGFP (1:1000, chicken from abcam ab13970), α-β-Galactosidase (1 :500, rabbit; Rockland immunochemicals Inc) and α-H3K9-tri-methylation (1:2000, #07-523 Millipore). The GFP recovery experiments were done as previously described in (17). *lacZ* expression assays were carried out using X-gal overnight staining as described in (18), except that ovaries were fixed for 6 min.

### Microscope analysis and image treatment

Immunostaining analysis was performed on a LEICA SP5 confocal microscope. GFP was viewed in whole-mount ovaries using the LEICA SP5 confocal microscope and analyzed using ImageJ software. 3D reconstruction was carried out using the Imaris software.

### qRT–PCR analysis

First strand cDNA was obtained by using random primers on Trizol-extracted total ovarian RNA from 2- to 3-day-old flies. Quantitative PCR was performed using Roche FastStart SYBR Green Master on the LC480 on two independent insertions for each transgene. Steady-state RNA levels were calculated from the threshold cycle for amplification using the 2^−Δ Δ ^*^C^_T_* method (21). *rp49* was used for the normalization. Average levels and standard deviations were calculated from at least four biological replicates according to (21). In RNAi experiments, fold enrichments correspond to the comparison with a *pGgIds*/RNAi-*white* control sample.

Primers for qRT–PCR analysis were

rp49_for: 5′-GACGCTTCAAGGGACAGTATCTG-3′

rp49_rev: 5′-AAACGCGGTTCTGCATGAG-3′

gfp_for : 5′-TACCTGTCCACACAATCTGC-3′

gfp_rev : 5′-ATCCATGCCATGTGTAATCC-3′

HeT-A_for : 5′-CGCGCGGAACCCATCTTCAGA-3′

HeT-A_rev : 5′-CGCCGCAGTCGTTTGGTGAGT-3′

TART_for : 5′-TTTTCCGGATCCAAGTGAAC-3′

TART_rev : 5′-TCTGGTCGTCGGAAGTTGTT-3′

I _for : 5′-CAAAAACAACAATACCGCTAAT-3′

I _rev : 5′-AGCAGGTTGCCGTCTCTTGTA-3′

roo_for: 5′-CGTCTGCAATGTACTGGCTCT-3′

roo_rev: 5′-CGGCACTCCACTAACTTCTCC-3′

stalker4_for: 5′-TTTGGAAGATTACCAAGGCAGTTCGC-3′

stalker4_rev: 5′-GGATCTAACTTATGACCCGATTCGTTCC-3′

## RESULTS

### Engineered transgenes functionally demonstrate the ability of the germline to silence *Idefix*

**Figure 1.**
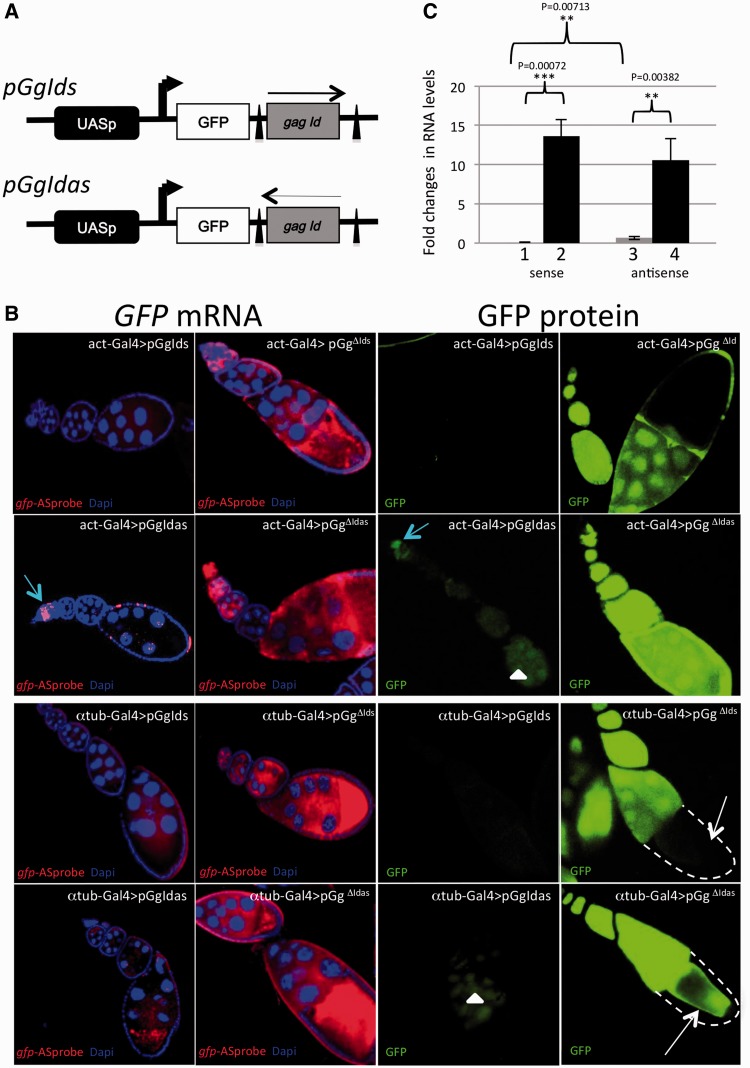
Structure and silencing of *pGgIds* and *pGgIdas* transgenes. (**A**) Structure of *pGgIds* and *pGgIdas*: The minimal promoter and the Gal4 target sequences (UASp), the *gfp* reporter gene (GFP) and 419 bp of the *Idefix gag* coding region [from nt 1003 to 1422 (*gag Id*)] are indicated. Arrows indicate the orientation of the *Idefix* sequence, which was inserted in either the sense (*pGgIds*) or anti-sense orientation (*pGgIdas*). The two FRT sites flanking the *Idefix* fragment are indicated as black triangles. The transcription initiation sites between UASp and GFP are indicated by arrows. (**B**) Representative images of ovariole expression of transgenic constructs with the *Idefix* sequence in either the sense or anti-sense orientation (*pGgIds* and *pGgIdas,* respectively) or without the *Idefix* sequence (*pGg^Δ^^Ids^* and *pGg^Δ^^Idas^*). Two drivers were used: the ubiquitous driver *actin-Gal4* (rows 1 and 2) and the germline driver *αtub-Gal4* (rows 3 and 4). *gfp* expression is presented at the mRNA (left, red) and protein (right, green) levels. Ovarioles are oriented with the germarium to the top or left. DNA was counterstained with DAPI (first and second columns, blue). Using both drivers, the transgene carrying the *Idefix* sequence in the sense orientation (*pGgIds*) exhibits undetectable levels of GFP mRNA and protein (columns 1 and 3). Using *αtub-Gal4* and *act-Gal4* drivers, the transgene carrying the *Idefix* sequence in the anti-sense orientation (*pGgIdas*) exhibits a low GFP expression in the nurse cells (white arrowheads) while a relatively strong GFP expression is detected only in the germarium (blue arrow) with the *act-Gal4* driver. Upon excision of the *Idefix* fragment (*pGg^Δ^^Ids^* and *pGg^Δ^^Idas^*), strong derepression of both *gfp* mRNA and protein is observed for both transgenes using both drivers. No *gfp* expression in somatic cells is observed when *αtub-Gal4* is used (dashed line and white arrow in fourth column). (**C**) Quantitative RT–PCR analysis of steady-state levels of transcripts encoded by *Idefix*-sensors in ovaries. *pGgIds insertion s1* (1), *pGg^Δ^^Ids^ insertion s1* (2), *pGgIdas insertion as1* (3) and *pGg^Δ^^Idas^ insertion as1* (4) driven by the *αtub-Gal4.* Experimental quadruplicates were carried out using *gfp* specific primers (see primer sets in ‘Materials and Methods’ section, mean ± SD and standard *P < 0.05, **P < 0.01, ***P < 0.001) and normalization, see ‘Materials and Methods’ section.

We developed a transgenic model that provides a convenient read-out to study piRNA-linked repression of the retrotransposon *Idefix* in the germline. The *Idefix*-sensor transgenes contain a *gfp* reporter gene (G) linked to a fragment of the *Idefix gag* gene in either a sense (*gIds*) or an anti-sense (*gIdas*) orientation (Figure 1A). The *Idefix* fragment is flanked by FRT sequences. Expression of the transgene is under the control of the germline *UASp* promoter (*p*). Six independent lines each carrying a single insertion were generated for each transgene (*pGgIds* and *pGgIdas*) using *P-*element transformation and the chromosomal mapping of each insertion was established. For each of the 12 transgenic lines, *pGgIds* and *pGgIdas* were expressed using two different *Gal4* drivers, i.e. the ubiquitous *actin-Gal4* (Figure 1B, rows 1 and 2) and the germline-specific *α4tubuline-Gal4 (αtub-Gal4*) (Figure 1B, rows 3 and 4). For the two drivers and the six *pGgIds* insertions tested, GFP was not detected, both at neither the RNA nor protein levels, in the germline throughout oogenesis (an example is presented Figure 1B, first and third columns, rows 1 and 3). For *pGgIdas,* faint GFP expression was generally observed in the nurse cells with both drivers (Figure 1B, first and third columns, rows 2 and 4), except for early stages of oogenesis within the germarium during which marked GFP protein and RNA signals were always detected (Figure 1B, row 2). When the *Idefix* sequence was excised upon recombination between the flanking FRTs, GFP RNA and protein signals were high for all the resulting *pGg^Δ^^Id^* transgenes (Figure 1B, second and fourth columns) indicating that the *Idefix*-sequence in the fusion transcript is responsible for GFP repression in the germline.

Quantitative RT–PCR with *gfp*-specific primers to measure the expression of the *gfp-Idefix* fusion transgenes confirmed that *Idefix* is a target of the germline repression (Figure 1C, histograms 1 and 3). In addition, *pGgIdas* displayed 2-fold higher expression than *pGgIds* (*P*-value = 0.0071). After *Idefix* was flipped out, the amount of RNA produced by *pGgIds* and *pGgIdas* increased >13-fold (*P*-value = 0.0007) and 10-fold (*P*-value = 0.0038), respectively (Figure 1C, histograms 2 and 4).

We conclude that the *Idefix*-based reporter constructs *pGgIds* and *pGgIdas* are targets of germline repression and that this repression is correlated with the presence of *Idefix* sequences within the transgenes.

### Mutations in components of the piRNA pathway impact *Idefix*-sensor silencing with different temporal requirements

**Figure 2.**
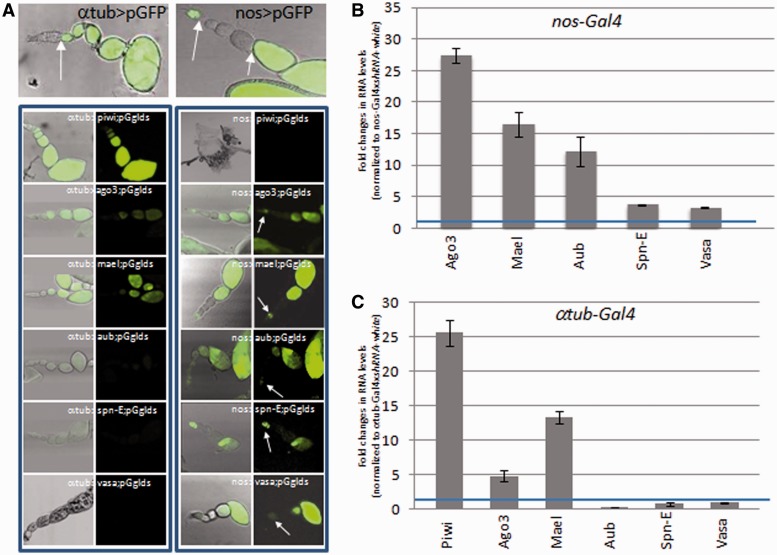
piRNA pathway components show different temporal requirements for *Idefix* silencing during *Drosophila* oogenesis. (**A**) GFP fluorescence is shown as a readout for *pGgIds* silencing release using two drivers, *αtub-Gal4* (left column) and *nos-Gal4* (right column) and RNAi constructs targeting *piwi, ago3, mae1, aub, spnE* and *vasa* transcripts. The expression profile of both drivers is presented at the top of each column using a GFP reporter construct (pGFP) and arrows delimit stages where these drivers are active. Ovarioles are oriented with the germarium to the top or left. *nos* > *piwi RNAi* results in atrophic ovaries. (**B** and **C**) *gfp* RNA was quantified by qRT–PCR using *gfp*-specific primers upon RNAi expression targeting the indicated genes and normalized to RNAi against *white* (the blue line represents no enrichment, value = 1). The transgene *pGgIds* was driven with *nos-Gal4* (B) or *α tub-Gal4* (C) (*n* = 4 biological replicates. Error bars represent SEM). Two transgenic lines *pGgIds* (s1 and s6) were tested in this experiment.

We then addressed the nature of the silencing exerted on *Idefix*-sensors in the germline in particular with respect to the piRNA pathway. We made use of TRIP RNAi lines (22,23), driven by α*tub-Gal4,* to target *piwi*, *ago3*, *aub mael*, *spn-E* and *vasa* (24–26). Since most of the genes tested have been shown to be key regulators of germline development, the fact that RNAi-mediated knockdown of all of these genes resulted in sterility with more or less severe ovarian phenotypes indicated the validity of the RNAi lines (data not shown). *piwi*, *ago3* and *mael* knockdown released silencing exerted on *pGgIds* leading to GFP expression as soon as the α*tub-Gal4*driver was active from Stage 3 of oogenesis. Surprisingly this was not the case when *aub, spn-E* or *vasa* were targeted by RNAi since GFP expression was never recovered (Figure 2A, left)*.* Results of quantitative RT–PCR with primers specific for *aub*, *spn-E* and *vasa* indicated that transcripts for these genes were indeed strongly reduced in ovaries compared with controls (Supplementary Figure S1A). At the protein level, immunofluorescent staining showed that α*tub* >*aub-* and *>vasa-RNAi* combinations resulted in strong depletion of Aub and Vasa proteins, respectively, as of Stage 3 of oogenesis when the driver is active, but these proteins were easily detected at earlier stages, in particular in the germarium, when this driver is inactive (Figure 2, top left and Supplementary Figure S1B).

We next expressed the RNAi-constructs in the GSC present in the germarium up to Stage 2 using the *nanos-Gal4* (*nos-Gal4*) driver. This driver is subsequently inactive between Stages 3 and 6 of oogenesis and re-activated at later stages as shown in Figure 2A (top right). When knockdown of *ago3*, *mael*, *aub*, *spnE* and *vasa* was performed using the *nos-Gal4* driver, we found that the *Idefix*-sensors were derepressed and GFP expression recovered within the germarium and after Stage 6 but not between Stages 3 and 6 as expected according to *nos-Gal4* activity (Figure 2A, right panel).

To confirm these results, we used quantitative RT–PCR with *gfp*-specific primers. Under RNAi conditions leading to derepression of GFP at the protein level (*nos > ago3*, *mael*, *aub*, *spn-E* and *vasa*; α*tub > piwi*, *ago3* and *mael*), an increase in *gfp* RNA was also detected although in a lower amount in *nos > spn-E* and *>vasa* flies (Figures 2B and C). In contrast, the amount of *gfp* RNA was unchanged in α*tub > aub*, *spn-E* and *vasa* RNAi ovaries compared to controls, as was observed with GFP protein expression (Figure 2C).

Sensor de-repression in late egg chambers from *nos > aub* and not from α*tub > aub* flies could potentially be explained by a higher strength of the *nos-Gal4* driver leading to a higher efficiency in knocking down target genes during late oogenesis. To compare the efficiency of the two drivers, we expressed an *UASp-gfp* transgene (*pGg^Δ^^Id^*) driven by *nos-Gal4* or α*tub-Gal4* driver, and quantified the *gfp* RNAs produced. We found almost twice as much *gfp* RNAs in (p*Gg^Δ^^Id^*, α*tub-Gal4*) as in (*pGg^Δ^^Id^*, *nos-Gal4*) flies (Supplementary Figure S2) which indicates that *nos-Gal4* is weaker than α*tub-Gal4*.

**Figure 3.**
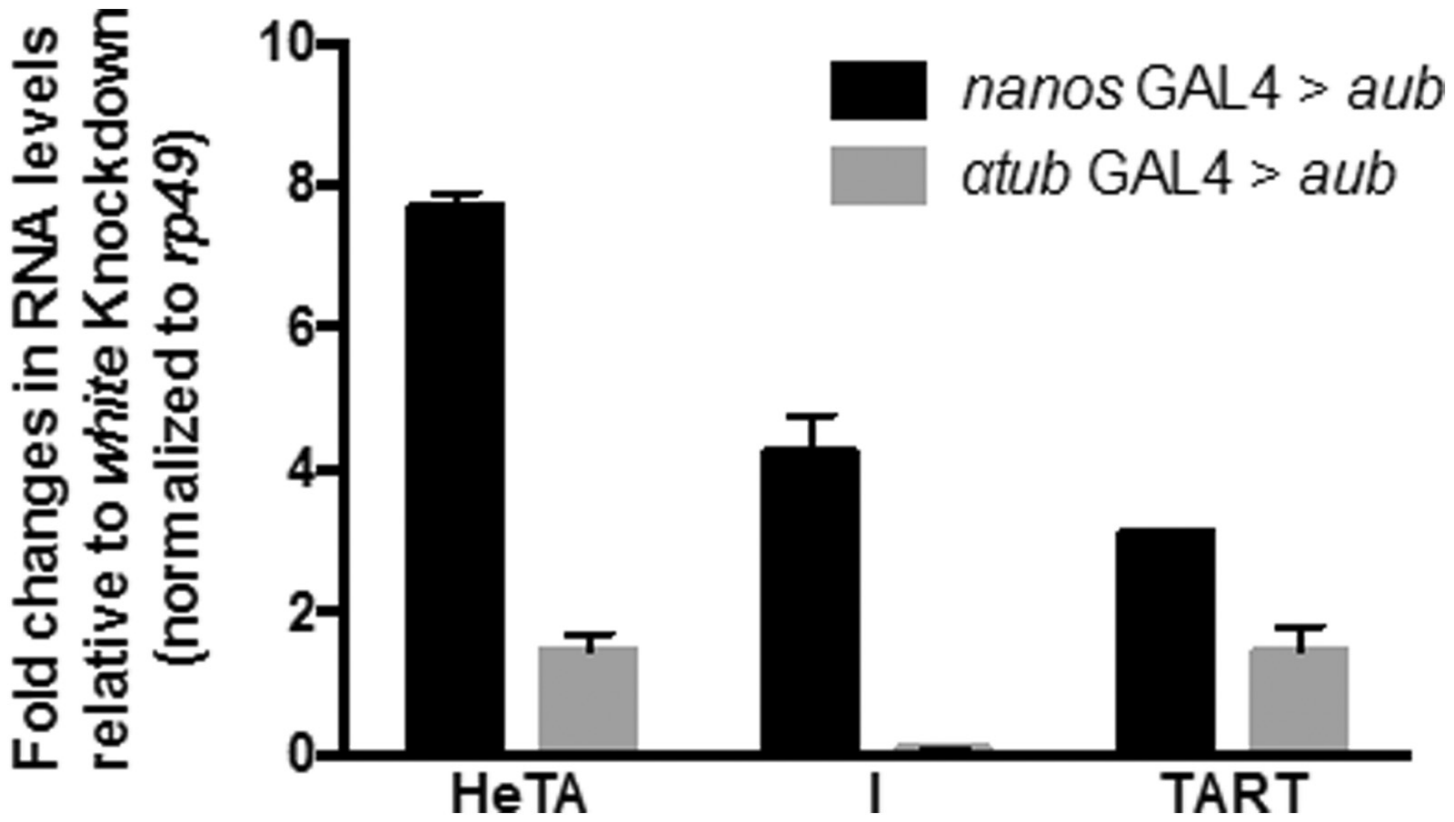
Knockdown of *aub* driven by either *nos-Gal4* or *αtub-Gal4* driver has different impact on germline transposon de-silencing. Relative expression levels of the indicated TEs upon knockdown of *aubergine* using either *nos*-Gal4 or *αtub-Gal*4 driver. Each knockdown is normalized to *white* knockdown controls and to *rp49*. *n* = 3 biological replicates. Error bars represent SD.

If the silencing exerted on *pGgIds* is released when knockdown of *aub* is driven by *nos-Gal4* and not α*tub-Gal4*, then, the silencing exerted on endogenous TE should also be differently affected in these mutant backgrounds. Quantitative RT–PCR was performed with specific primers targeting germline TEs: *HeT-A*, *I-*element, and *TART*. Similarly to the results obtained with *Idefix* sensors, a higher increase of *HeT-A*, *I* and *TART* RNAs was observed in *nos-Gal4* > *aub* than in *αtub-Gal4* > *aub* ovaries (Figure 3). These findings also indicate that knock down of *aub* after Stage 3 of oogenesis fails to release *HeT-A*, *I* and *TART* silencing.

Thus, our findings suggest that a spatio-temporal regulation of components of the piRNA pathway exists throughout oogenesis. Genes such as *aub*, *spn-E* and *vasa* are only necessary during the first stages of oogenesis up to Stage 3 to mediate silencing. Genes such as *piwi*, *ago3* and *mael* are needed to silence piRNA targets both at early stages, within the germarium, and constantly thereafter.

### Germline repression of *Idefix*-sensors is highly impaired in dividing germline cells in the germarium

**Figure 4.**
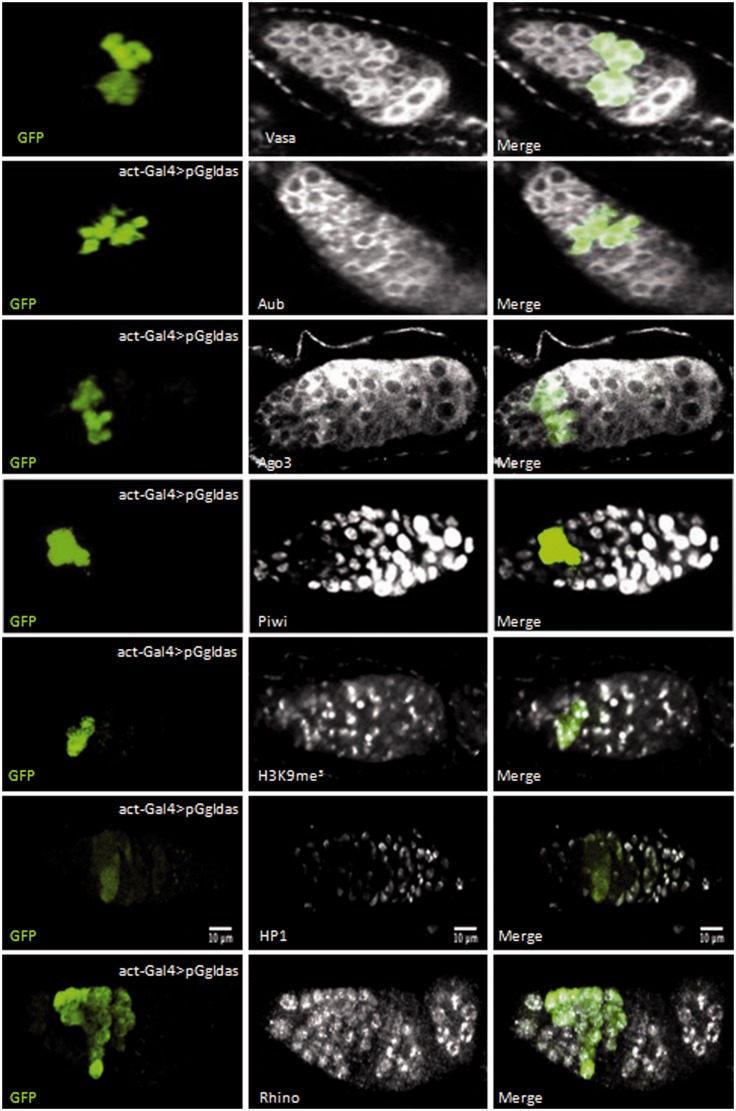
*Idefix* silencing is reduced in the dividing germline cysts of the germarium. Immunodetection of Vasa, Aub, Ago3, Piwi, H3K9me^3^, HP1 and Rhino proteins (white) and GFP (green) in germaria of ovaries expressing the *pGgIdas* transgene under the control of the *act-Gal4* driver. Germaria are oriented with anterior to the top or left. GFP signal is specifically observed in 4-, 8- and 16-cell germline cysts in which Piwi does not accumulate. Experiments were performed on *pGgIdas* lines as1 and as2.

Although piRNA silencing of *Idefix*-sensor transgenes is efficient in the germline nurse cells, we observed that a small patch of cells located at the anterior tip of the germarium presented GFP fluorescence in ovaries in which the *pGgIdas* transgene was under control of the *actin-Gal4* driver (Figure 1B, line 2). Co-immunofluorescence experiments revealed that GFP protein expressed from *pGgIdas* co-localized with the germline marker Vasa in the germarium (Figure 4, line 1). Upon examination of more than 100 germaria, co-localization of GFP and Vasa was only observed in dividing germline cysts of 4, 8 or 16 cells.

A possible explanation for the defect in silencing of antisense *Idefix*-sensors in dividing germline cysts is that the piRNA pathway is less efficient in these cells. Thus, we performed immunostaining experiments to examine the expression of major actors of the piRNA pathway. Aub- and Ago3-antibodies revealed that both proteins were expressed throughout the germarium including in cysts expressing GFP from the *Idefix*-sensors (Figure 4, lines 2 and 3). We then examined Piwi whose expression has been reported to be weak in the germarium (27,28). In contrast to Aub and Ago3, although the Piwi antibody revealed the presence of Piwi protein in GSCs at the very anterior end of the germarium, as well as later in germarial region 2b, Piwi protein was barely detected in dividing germline cysts expressing GFP from the *Idefix*-sensors in more than 150 germaria examined (Figure 4, line 4 and Supplementary Movie S3).

To further characterize these cells, we also examined the presence of H3K9me3 and HP1 in early germline cells. Both have been recently reported to be present in Repressive Chromatin Centers (RCCs) which are required in the germline for piRNA production (29). We performed immunostaining experiments with an H3K9me3 antibody and observed, as Rangan *et al.* (29), a signal in prominent and discrete foci from the cystoblast to the late stages of germarial development (Figure 4, line 5). In contrast, HP1 signal was only detected at a low level at early stages in the germarium including in cells in which *pGgIdas* repression is inactive (Figure 4, line 6 and Supplementary Movie S4). Finally, we examined the expression of the Drosophila HP1 homolog, Rhino, required for piRNA cluster transcription and piRNAs production (30). Immunostaining experiments revealed that Rhino is uniformly expressed all along the germarium including the dividing cysts (Figure 4, line 7). Thus, although Aub, Ago3 and Rhino are expressed and H3K9me3 detected in RCCs, silencing of the *Idefix-*sensors is weak in dividing germline cysts that exhibit low levels of Piwi and HP1 proteins.

### A post-transcriptional control represses Piwi in the dividing cysts

**Figure 5.**
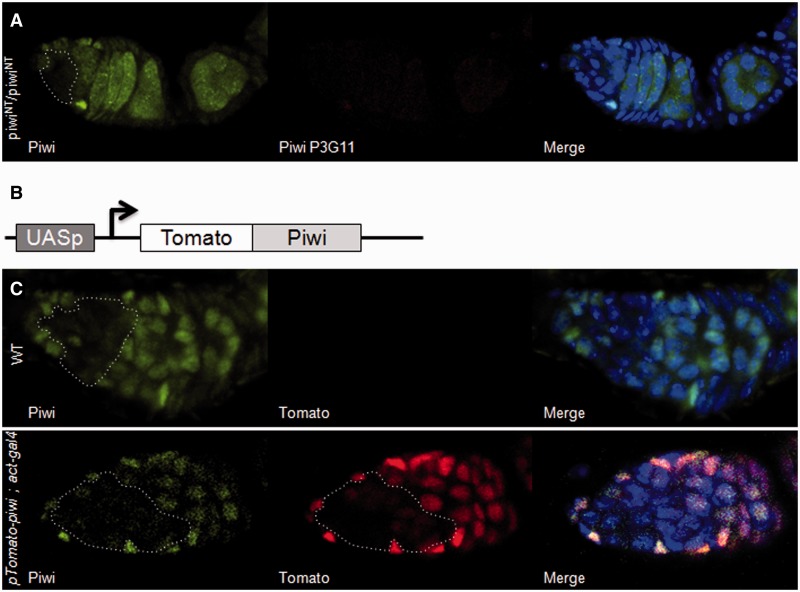
The weak expression of *piwi* in the dividing cysts is due to a post-transcriptional and/or translational repression. (**A**) Immunodetection of Piwi in germaria of *piwi^NT^* mutant ovaries. Polyclonal anti-Piwi (left, green) is directed against the central part of the Piwi protein. Monoclonal anti-Piwi Nter P3G11 (red) is directed against the amino-terminal part of Piwi which is deleted in the *piwi^NT^* mutant. As expected for a *piwi^NT^* mutant, no signal is revealed by the monoclonal antibody. In constrast, Piwi proteins are detected by the polyclonal antibody in the germ cells except within the dividing cysts. (**B**) Molecular structure of the *pTomato-piwi* transgene. (**C**) Tomato fluorescence is shown as a readout for *pTomato-piwi* expression. Endogenous Piwi proteins are detected using the polyclonal anti-Piwi (right). The expression profile of endogenous and transgenic Piwi is presented on the left and middle, respectively. When no driver is introduced in the line (upper panel), no Tomato fluorescence is detected in the germline. When *pTomato-piwi* is driven by *actin-Gal4* in the germline (lower panel), Tomato fluorescence is revealed in the germline but remains undetected within the dividing cysts. DNA was counterstained with DAPI (blue).

Piwi localization in germline and follicle cells is predominantly nuclear. We examined Piwi localization in mutant flies, *piwi^NT^*, in which Piwi nuclear accumulation is prevented (31). A polyclonal Piwi antibody revealed Piwi deleted from its N-terminus in the cytoplasm. The signal was intense in all the germline with the exception of the dividing cysts, where only a very faint signal similar to the wild-type Piwi signal could be detected (Figure 5A, left) (31). Thus, endogenous Piwi expression is highly reduced in dividing cysts.

We then speculated whether Piwi signal could be recovered in these cells if *piwi* was placed under the control of the heterologous promoter UASp. We established a transgenic line with a *piwi* cDNA fused to the *Tomato fluorescent protein* gene under the control of UASp (Figure 5B and ‘Materials and Methods’ section). When driven by *actin-Gal4* to achieve germline expression, Tomato-Piwi fluorescence was detected, like endogenous wild-type Piwi, in the germ cells except the dividing cysts, in which the signal was faint (Figure 5C). Given that *actin-Gal4* is active in these cells as illustrated Figure 4, we concluded that post-transcriptional and/or translational repression of Piwi exists during this short window of germline development.

### Germline repression of a transgenic model mimicking the *P*-element repression is also impaired in dividing germline cells in the germarium

**Figure 6.**
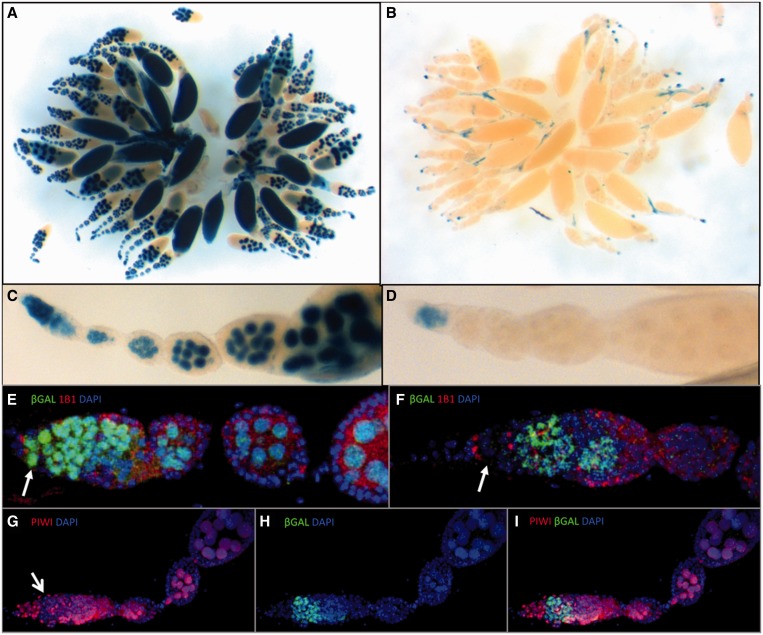
*Trans*-silencing in the female germline associated to *P*-element repression is strongly weakened in dividing cysts in the germarium. (**A**, **C**, **E**) Ovaries from *BC69* females carrying one copy of a *P-lacZ* enhancer-trap inserted into the *vasa* gene. (**B**, **D**, **F–I**) Ovaries from females produced by the cross of *BC69* males with *RS3* females, which carry a *P-FRT-white* silencer transgene in the TAS of the *3R* chromosomal arm. (**A–D**) X-gal staining. *BC69* females carrying a *P-lacZ* transgene which contains the sequence for a NLS (Nuclear Localization Signal) fused to the *lacZ* coding sequence shows nuclear staining (A, C). βgalactosidase is strongly reduced in the presence of the TAS-associated silencer transgene except in germaria (B, D). (**E**, **F**) Double immunostaining of a germarium for 1B1 (red), which marks the spectrosomes of GSCs (arrows), and β-galactosidase (green). DNA was counterstained with DAPI (blue). In the presence of the TAS-associated silencer transgene (F), β-galactosidase expression is incompletely repressed in the region of the dividing germline cysts just next to the GSCs. (**G–I**) Double immunostaining of a germarium for Piwi (red) and β-galactosidase (green). DNA was counterstained with DAPI (blue). Incomplete repression correlates with weak Piwi staining in the germarium (marked by an arrow in G).

To determine whether the silencing defect in dividing germline cells is unique to *Idefix*-sensors, we investigated the silencing exerted on another TE, the *P*-element, a DNA transposon. *P* repression has been shown to be primarily established by *P* copies inserted in sub-telomeric heterochromatin loci (TAS) (32), which are strong piRNA-producing loci (2). Indeed, a single defective *P*-element inserted in the TAS of the *X*-chromosome can repress the activity of 80 *P* copies *in trans* (33). A transgenic model that mimics *P* repression was previously established using *P-lacZ* enhancer-trap transgenes (34). Telomeric *P-lacZ* inserted in TAS can repress a homologous *P-lacZ* in *trans,* irrespective of the genomic location of the latter (35,36). This *trans*-silencing effect (TSE) is restricted to the germline. Incomplete repression is frequently observed in the germarium and occasionally during later stages of oogenesis which results in an ‘on’ or ‘off’ target expression between egg chambers (variegation). However, we found that with some combinations of silencer-target transgenes, incomplete repression was strictly restricted to the germarium. The *BC69 P-lacZ* enhancer trap (18) for example, showed strong *lacZ* expression in all the germ cells of the ovarioles, including the GSCs (Figure 6A, C and E). When this transgene was submitted to *trans*-silencing mediated by a maternally-inherited telomeric *P-FRT-white* transgene (*RS3*) inserted in the TAS of the *3R* chromosomal arm, complete repression was observed at all stages except for in the germarium (Figure 6B, D and F). Co-immunostaining experiments using 1B1, a marker for the GSC spectrosome and the branched fusomes of dividing germline cysts, and anti-β-galactosidase antibodies, showed that repression occurs in GSCs and at later stages outside of the germarium, but that dividing germline cysts in the middle of the germarium escape strong repression (Figure 6F). Finally, co-immunostaining experiments, using anti-Piwi and anti-β-galactosidase antibodies, revealed that impaired repression in germline cysts in the germarium correlated with low Piwi staining (Figure 6G–I).

Overall, our results indicate that two different types of TEs (the *Idefix* retrotransposon and the DNA-based *P*-element) may escape piRNA-mediated repression during oogenesis in a window when germline cysts divide within germarial region 2a. This stage-specific release from piRNA-mediated repression is probably made possible, at least in part, through reduced Piwi expression at these stages. We propose to call this region the ‘Piwiless pocket’ or Pilp.

## DISCUSSION

### *Drosophila* female germline cells have the ability to repress *Idefix via* the piRNA pathway even though the *Idefix* promoter is not active in these cells

The *Idefix* promoter is active in follicular somatic cells and inactive in other somatic cells and in the germline (37), but *Idefix* repression mechanisms exist in all three tissue types. In follicular cells, the linear piRNA pathway is responsible for silencing *Idefix*, while in other somatic tissues we have shown that PTGS and TGS cooperate to silence *Idefix* reporter transgenes (17). We now show that the presence of an *Idefix* fragment allows silencing of engineered transgenes in the germline *via* the piRNA pathway. Since numerous *Idefix* copies are present in heterochromatic piRNA clusters (2,38), which produce piRNAs complementary to *Idefix* mRNAs, these are likely responsible for specific recognition of the transgene transcripts (37). Thus, the huge reservoir of multiple TEs constituting heterochromatin may protect the germline by anticipating sudden activation of a TE promoter that previously displayed somatic specificity, or from newly-incoming homologous TEs that have invaded the species by horizontal transfer (39).

### *Idefix* piRNA-mediated silencing involves two categories of genes with different temporal requirement throughout oogenesis

Since the silencing that targets *Idefix*-reporters in the germline is removed once the targeted sequence is lost, we used these genetic tools to dissect spatio-temporal requirements for piRNA-mediated silencing in *Drosophila* germline.

In contrast to previous studies in which components of the piRNA pathway in the germline were disrupted by expressing RNAi using *nos* or *MTD* driver that are both active in the GSC (40–42), we decided to compare piRNA-mediated silencing when two drivers whose activity patterns differ during oogenesis, *nos* and *αtub*, are used (Figure 2A, upper panel). This comparison allowed the identification of two categories of piRNA components distinguished according to their temporal requirement. The activity of one of them includes Piwi, Ago3 and Mael and is needed in germline cells to repress *Idefix*-sensors continuously during oogenesis. Indeed depletion of these proteins within the germarium and after Stage 6 of oogenesis as a result of *nos-Gal4* activity, or as of Stage 3 of oogenesis following *αtub* activity, leads to sensor-transgene desilencing (Figure 3, rows 1, 2 and 3). Another category of proteins, including Aub, Vasa and Spn-E, is required for silencing only in the earliest stages of germline development, before Stage 3, for efficient TE silencing all along the ovariole. If their depletion occurs after the germarium stage following *αtub* driver activity, *Idefix*-sensor silencing is never released. *Idefix* sensors are suitable tools to follow temporal requirements of piRNA pathway components because of their easy GFP read-out. However, we also reported that the silencing of endogenous TEs, *HeT-A*, *I-*element and *TART*, is differently affected by *nos*> and *atub* > *aub* knock down. As observed with *Idefix*-sensors, these TEs remain silenced when *aub* is depleted after the germarium stage whereas their silencing is released if *aub* depletion occurs as from the GSC. Nevertheless, the sensitivity of silencing to piRNA component knocked-down may differ between TEs. Several observations made by others indicated that loss of Spn-E has more severe effects on several endogenous TEs than loss of Aub (43,44). Here, we found that *Idefix*-sensor release is weaker when Spn-E is depleted than Ago3, Aub or Mael. Overall, our observations are further evidence of the complexity of TE silencing in the germline.

Interestingly, RNAi knockdown of *aub, vasa* and *spn-E* genes with both the early *nos-Gal4* and the later *αtub-Gal4* driver resulted in sterility. This is consistent with the axis patterning defects previously characterized for mutations in these genes (45). Hence, loss of function of this category of genes after Stage 3 of oogenesis impairs fecundity, but does not impair *Idefix-*sensor silencing nor the silencing of some TEs tested in this study such as *HeT-A*, *I-*element and *TART* repression. This suggests that defects in oogenesis due to *aub, vasa* and *spn-E* RNAi-depletion after Stage 3 might not be caused by transposition of TEs.

The perinuclear structure, the nuage, is involved in TE silencing (46,47). Many genes have been reported to be required for its assembly including *aub*, *vasa* and *spn-E*. The early requirement for these genes for *Idefix*-sensor silencing might be explained by their role at the initiation phase of nuage formation. Immunostaining experiments performed with an Ago3 antibody revealed that, if *aub* and *vasa* are knocked down after Stage 3 of oogenesis (*αtub-Gal4* driving RNAi), the nuage is still observed around the germline nuclei of late-stage egg chambers (although fainter with *vasa* RNAi, see Supplementary Figure S5). Thus, it is conceivable that *aub*, *vasa* and *spn-E* function at the initiation phase of nuage formation. Once established, the nuage (or a sufficient structure) may persist throughout oogenesis independently of the factors that initiated its assembly, and thereby assure TE silencing. Alternatively, could an heritable silenced state be established on TEs before Stage 3 when *aub*, *vasa* or *spn-E* are active owing to the non-functional *αtub* driver? Why would this silenced state not be established when *piwi*, *ago3* and *mael* are knocked down by the same driver? Further investigations are needed at this stage to understand the roles played by these components during oogenesis.

Our present data raise another important issue. In the ping-pong cycle of piRNA production, Ago3-bound-piRNAs have been shown to pair with Aub-bound piRNAs (2). Thus, the ping-pong cycle would be expected to collapse when either of the two PIWI proteins is depleted. However, *Idefix*-sensors are derepressed in *αtub* > *Ago3*-RNAi and not in *αtub* > *aub*-RNAi ovaries. In their study, Brennecke *et al.* (2007) showed that self-complementarity also occurs for both Aub- and Ago3-bound piRNA 5′-ends. Furthermore, Zhang et *al.* reported that both heterotypic and homotypic piRNA ping-pongs exist (48). The findings of our study support the existence of homotypic Ago3-Ago3 ping-pong for *Idefix*-sensor repression in ovaries depleted for Aub after Stage 3 of oogenesis. Taken together, our results suggest that *aub*, *vasa* and *spn-E* are necessary for an initial step of the piRNA pathway, possibly nuage formation, while *piwi*, *ago3* and *mael* are required continuously for piRNA-mediated silencing.

### TEs can escape host piRNA silencing in the ‘Piwiless pocket’ during early oogenesis

A detailed analysis of the repression exerted on *Idefix*-sensor transgenes throughout oogenesis revealed that its repression is partially released during a short window within the germarium corresponding to cystocytes undergoing mitotic divisions to form the interconnected 16-cell germline cysts. While *pGgIdas* silencing is largely absent in germline cysts of 4–16 cells, *pGgIds* repression is never released in these cells. Since silencing of *pGgIdas* is weaker than that of *pGgIds* as revealed by occasional expression along the ovariole, it is likely that *pGgIdas* expression in the dividing germline cysts corresponds to a decrease in the efficiency of the silencing pathway. Accordingly, we found that, although *aub* and *ago3* are both constantly expressed in the germline, *piwi* expression is low in dividing germline cysts, but is clearly detected earlier in GSCs, and later, from region 2b of the germarium onwards. Our data further indicated that this decrease in Piwi is not due to a transcriptional regulation exerted on its own promoter but rather to a post-transcriptional repression exerted specifically in these cells. To elucidate at the molecular level why the Piwi protein level is lower even when it is expressed from the *pTomato-piwi* transgene, we are currently investigating other differences in piRNA-depending factors within and outside the dividing cysts.

If, as suggested above, piRNA silencing is weak in dividing germline cysts, *Idefix* would not be the only TE whose repression is affected in these cells. In fact, we found that germline cysts of 4–16 cells are also impaired for a homology-dependent silencing of the *P*-element, a transposon whose repression is established in the germline primarily by *P* copies inserted in subtelomeric piRNA-producing heterochromatin. *P*-element repression was found to be efficient earlier in GSCs and cystoblasts. Thus, although our experiments were performed on transgenes, our data suggest that repression of both the *P*-element and *Idefix* is impaired specifically in dividing germline cysts in which Piwi expression is low (the ‘Piwiless pocket’ or Pilp). It has to be noticed that, in a recent article, Shpiz *et al.* observed similar results with the retroelement *HeT-A* (44). Endogenous *HeT-A* copies as well as *HeT-A*-LacZ constructs are silenced in wild-type ovaries by the piRNA-mediated pathway. In homozygous *piwi*/*piwi* mutants, their expression is recovered along the whole ovariole. Interestingly, in heterozygotes *piwi*/+ females, their silencing is partially released giving rise to *HeT-A* or *HeT-A-LacZ* expression in germarial cysts only. The restricted expression of *HeT-A* in these cells could be due to an increased weakness of the piRNA pathway resulting from the heterozygous *piwi*/*+* background in the Pilp, as seen with *P* and *Idefix* transgenes.

Since in the Pilp we observed RCCs, which present the H3K9me3 repressive mark and HP1a, but in a lower amount than in the rest of the ovarioles, it is possible that, in the dividing cysts, the absence of Piwi prevents H3K9me3 from recruiting HP1a. The activity of the piRNA clusters within RCCs would then be affected and TE silencing released or decreased. Further characterization of the Pilp will certainly help address more precisely how Piwi functions in germline transposon silencing, in particular in these few cells of the germarium.

Overall, our data strongly suggest that a window exists during early stages of oogenesis, the Pilp, during which TEs within germline cells can escape from host restraint and transpose. The fact that this window corresponds to dividing germline cysts may provide advantages for both TE regulation and genome dynamics. Since this phenomenon occurs in the germline, transmission of new, potentially beneficial, TE genomic insertions to the next generation is assured. Conversely, since TE derepression does not occur in GSCs, lethal or highly damaging mobilization events will not be produced in these cells and the potential to produce new viable germline cysts will be maintained. In addition, the existence of a Pilp in the germline may explain how sporadic reactivation of TEs occurs (49,50). Overall, these findings provide insight into the reasons for the highly successful invasion of genomes by TEs.

## SUPPLEMENTARY DATA

Supplementary Data are available at NAR Online. 

## FUNDING

Grants-in-aid from Clermont University, the Centre National pour la Recherche Scientifique (CNRS), the Institut National pour la Santé et la Recherche Médicale (INSERM); This work was supported by the Ministry of Education (to J.D.); the Région Auvergne [Nouveau chercheur to E.B.]; and European Union (to P.P.); and Agence pour la Recherche contre le Cancer (ARC to J.D.); the Ligue régionale contre le Cancer (to C.V.). Funding for open access charge: INSERM U1103, CNRS 6293.

*Conflict of interest statement*. None declared.

## Supplementary Material

Supplementary Data
