# Supplementary material for: Spatio-temporal requirements for transposable element piRNA-mediated silencing during Drosophila oogenesis
Source: Nucleic Acids Res. 2013 Nov 27;42(4):2512–24. doi: 10.1093/nar/gkt1184 (PMC3936749; doi:10.1093/nar/gkt1184)
Supplement: Supplementary Data [file supp_42_4_2512__index.html]

Spatio-temporal requirements for transposable element piRNA-mediated silencing during Drosophila oogenesis — Supplementary Data 

# Spatio-temporal requirements for transposable element piRNA-mediated silencing during *Drosophila* oogenesis

## Supplementary Data

files

**Files in this Data Supplement:**

- Supplementary Data - doc file
- Supplementary Data - mpg file
- Supplementary Data - mpg file
