# Supplementary material for: Spatio-temporal requirements for transposable element piRNA-mediated silencing during Drosophila oogenesis
Source: Nucleic Acids Res. 2013 Nov 27;42(4):2512–24. doi: 10.1093/nar/gkt1184 (PMC3936749; doi:10.1093/nar/gkt1184)
Supplement: Supplementary Data [file supp_gkt1184_Suplementary_Figures_1_2-3_6.doc]

**Supplementary Figure S1**

**Supplementary Figure** **S1**. Controls of the RNAi activity **(A)** For each RNAi background, RNAs were quantified by qRT-PCR using specific primers addressed against the knock-down gene (red panels). They were compared to the corresponding RNAs amplified from a RNAi line against *white* (blue panels respectively). The transgene *pGgIds* was driven with *αtub-Gal4.* Sequences of the primers used are given in supplementary data. (**B**) *αtub>aub- and vasa*-RNAi shows an apparent complete depletion of Aub and Vasa proteins as soon as the driver is active in germ cells from stage 3 of oogenesis. The studied lines are *pGgIds* sensor lines. The mutated genotypes are indicated in white. Antibodies used for immunostaining are indicated in pink. The third column displays an enlargement of the regions squared in column two.

**Supplementary Figure S2**

**Supplementary Figure S2** : The *αtub-Gal4* driver is stronger than the *nos-Gal4* driver. *gfp* RNAs were quantified by qRT-PCR using *gfp*-specific primers in ovaries expressing nos>*pGg^ΔIds^*, and αtub>*pG^ΔIds^*, normalized to *rp49* and reported to *pGg^ΔIds^* not driven (n=4 biological replicates. Error bars represent SD).

**Supplementary Figure S5**


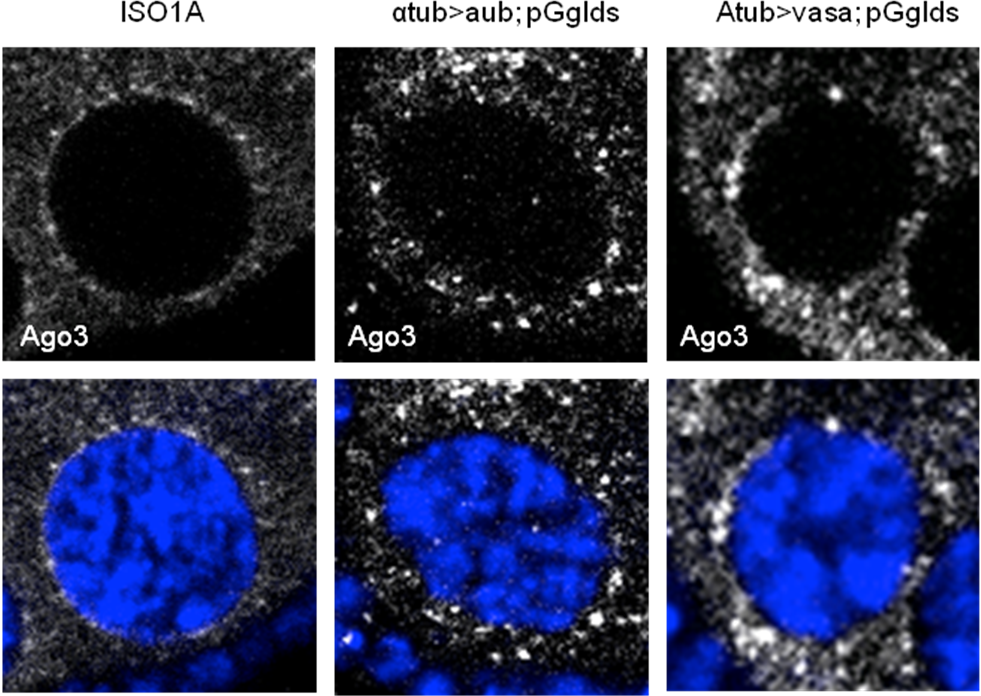


**Supplementary Figure S5**. Late depletion of germ line Aub and Vasa does not disrupt nuage formation.

Ago3 immunofluorescent staining of the perinuclear structure called the nuage in the nurse cells. Ago3 is observed around the nuclei of the Iso1A nurse cells taken as positive control (left) as well as in germ cells depleted for Aub and Vasa ( α*tub*>*aub*- and *vasa*-RNAi lines, middle and right respectively). DNA is shown in blue (Hoechst).

**Legends to Supplementary Movies**

Figure S3. Immunocytochemical analysis of Piwi (red) protein in the germarium of a *pGgIdas* transgenic line (GFP signal in green). The GFP signal is observed in germ cells where Piwi expression is low.

Figure S4. Immunocytochemical analysis of HP1 (red) protein in the germarium of a *pGgIdas* line (GFP signal in green). The H3K9me3 signals are observed in prominent foci. DAPI is indicated in blue.

**Primers for qRT-PCR analysis were:**

spnE_for: 5’-CGTTAATGACACCGGAAAAC-3’

spnE_rev: 5’-GATGGAGCTCTCAGCAATGT-3’

vasa_for: 5’-AGCTTGCTCTGATGTGAAGC-3’

vasa_rev: 5’-ACAATGGTGCCATCTGCTTG-3’

aub_for : 5’-ATTTTGTCCGATGCTATTCG-3’

aub_rev : 5’-ACATCGTCAATGCGATAGGT-3’
